# Hematology and serum biochemistry of free-range brown-throated sloths in two urban areas in Brazil

**DOI:** 10.14202/vetworld.2020.2033-2038

**Published:** 2020-09-30

**Authors:** Kissia Ferreira Pereira, Ita de Oliveira e Silva, Fernanda de Fátima Rodrigues Silva, Vinicius Herold Dornelas e Silva, Carla Soraia Soares de Castro, Vanner Boere

**Affiliations:** 1 Postgraduation Program at Animal Biology, Universidade Federal de Viçosa, Viçosa, Minas Gerais, Brazil; 2 Institute of Humanities, Arts and Sciences of the Universidade Federal do Sul da Bahia, Campus Jorge Amado, Itabuna, Bahia, Brazil; 3 Postgraduation Program at Ecology and Environmental Monitoring, Department of Engineering and Environment, Universidade Federal da Paraíba, Rio Tinto, Paraíba, Brazil

**Keywords:** order pilosa, physiology, public square, urban fauna, wildlife

## Abstract

**Background and Aim:**

The brown-throated sloth (*Bradypus variegatus*) is widely distributed in three biomes: The Amazon, Atlantic Forest, and Caatinga. Some subpopulations are isolated in urban areas in Brazil, usually in squares and small woods. Due to the scarcity of reference values, an investigation was carried out on the hematology and blood biochemistry of brown-throated sloths from urban areas.

**Materials and Methods:**

Blood was collected by venipuncture in the femoral vein from 19 brown-throated sloths for hematological and biochemical analyses, living in two municipalities; Teófilo Otoni (TO) (Minas Gerais State) and Rio Tinto (RT) (Paraíba state), in the Atlantic Forest biome, Brazil. The samples were analyzed in specialized veterinary laboratories using automatic cell counters and slide fixation staining methods.

**Results:**

The two subpopulations of brown-throated sloths had no significant differences in most hematological values, with the exception of a higher leukocyte concentration (p<0.01) in the RT sub-population. The difference in leukocyte concentration suggests an idiosyncratic effect, as the animals were healthy and the stress of the capture was acute, not chronic. The concentrations of urea, creatinine, and alkaline phosphatase (ALP) were similar (p<0.05) in brown-throated sloths from TO and RT. Likewise, the liver enzyme concentrations (ALP, alanine transaminase [ALT], and aspartate transaminase [AST]) did not differ between the two subpopulations (p<0.05).

**Conclusion:**

Compared to another study, brown-throated sloths from TO and RT have higher plasma concentrations of ALT and ALP, suggestive of a hepatic overload. Hematological and blood biochemical findings of TO and RT can be used as clinical reference values for brown-throated sloths living in an urban environment.

## Introduction

Improving knowledge of animal physiology is a constant task for wildlife researchers to determine benchmarks for assessing the health of wild animals [1]. In addition to capture stress, other factors can influence physiological patterns, such as the phylogenetic characteristics of subpopulations, the environment in which they live, and their life history [2]. It is necessary that a wide database of wild mammals be available to assist in the interpretation of the average, limits, and abnormal values of physiological indexes. The collection of wild animal data is primarily performed with animals in captivity and not free-range animals, in which values can be divergent [3].

Brown-throated sloths (*Bradypus variegatus*) have a wide geographical distribution, inhabiting forests in the Caatinga, Cerrado, Amazon, and Atlantic Forest biomes [4]. Although the International Union for Conservation of Nature classifies the animals as “Least Concern” for Conservation of Nature, the viability of some subpopulations of brown-throated sloths is threatened by the expansion of human activity, replacing forest areas with urban buildings, fields, and intensive agricultural plantations [5].

As a result of cultural aspects and the lack of management [6], in some regions, there are subpopulations of brown-throated sloths that live isolated in small wooded areas, often within urban squares [6,7]. Human activity brings other threats to brown-throated sloths, such as electrocution, risk of being run over, and attack by domestic animals in urban environments [8]. People take injured brown-throated sloths regularly to triage centers or veterinary hospitals. When performing clinical care, it is important to know the values of physiological standards in these animals. When humans and animals are restricted to small green space within urban areas, the proximity theoretically brings another threat; contagion by potential zoonotic pathogens. Brown-throated sloths have already been described as infected with sarcoptic mange (*Sarcoptes scabiei*), with *Leishmania* spp. [9,10], *Leiuris leptocephalus* [11], *Trypanosoma cruzi*, and *Trypanosoma leeuwenhoek* [12]. Sarcoptic mange, leishmaniasis, and trypanosomiasis have also been described in domestic animals and humans [13,14]. Referencing hematological data are important to support wildlife management studies to assess the health of the animals. Despite emerging diseases, arboviruses, and zoonosis [1] plaguing the habitats where brown-throated sloths live, little is known about the epidemiological role, if any, of the brown-throated sloths in the natural history of diseases that can affect them. Hematological and blood biochemical standards are a benchmark for assessing the health of sloths. Thus, there is a gap in studies on the ecology of sloths, despite being a widespread species in South America.

The aim of this study was to understand the ecology and biology of brown-throated sloths, aiming at defining the best practice of conservation medicine. Therefore, this work aims to describe the hematological and serum biochemical patterns in two subpopulations of brown-throated sloths living in an isolated urban area.

## Materials and Methods

### Ethical approval

The project was approved by the Animal Use Ethics Committee of the Federal University of Viçosa (authorization number 412013) and by the Biodiversity Authorization and Information System (authorization number 42162-3).

### Study area

The study was carried out in the Tiradentes and João Pessoa squares in the towns of Teófilo Otoni (TO) and Rio Tinto (RT), respectively. Both TO and RT are located in the Atlantic Forest biome, but the towns are located 1400 km apart. The Tiradentes square is located in the downtown area of TO, Minas Gerais, Brazil (17°51’52.73″S 41º30’29.48″O). TO is a town in the northeast of Minas Gerais, in the Mucuri Valley, considered the macro-regional center of commerce and has an estimated population of 140,000 inhabitants [15]. TO city has a tropical climate, characterized by a dry season [15]. The annual average temperature is 23°C, with dry and mild winters and rainy summers, and high temperatures [15]. During the study period, there were seven males, one female, and one very young brown-throated sloth.

The João Pessoa Square is located in the downtown area of RT, Paraíba (06°48′10″S 35°04′51″O). Its population, in 2012, was estimated by the IBGE at 23,431 inhabitants, spread over 466 km^2^ of area. The town of RT has a tropical climate and an average temperature of 25.9°C. The average annual rainfall is 1398 mm [15]. At the João Pessoa Square, during the study period, there were four adult females and 15 adult male brown-throated sloths.

### Collection and analysis

Eight adult sloths were captured in TO and 11 in RT, in October 2013 and October 2014, respectively. We have no way of knowing the precise age, but all the animals captured were considered adults due to their morphological characteristics. Using rappel techniques, the sloths were carefully removed from the trees by one of the researchers. The claws were immobilized using masking tape, and they were transported to the ground using a cotton bag and ropes. After manual containment without the use of anesthesia, 2–3 mL of blood was collected by venipuncture from the cephalic vein of the thoracic limb. A part of the sample was stored in tubes with ethylenediaminetetraacetic acid (EDTA; K3EDTA; Labor Import, São Paulo, Brazil) to analyze the blood count, and the other part in tubes without EDTA (Labor Import, São Paulo, Brazil) for biochemical analysis. Immediately after collection, the animals were released into the same tree in which they were captured. The samples were transported in cooled containers for the blood count and biochemistry analysis. Each collection procedure lasted a maximum of 30 min/sloth. Two veterinarians performed the venipuncture and clinically evaluated the sloths through visual inspection and palpation.

The blood counts of the samples of TO’s brown-throated sloths were performed at the Promed laboratory (TO, Minas Gerais, Brazil), and the RT samples were analyzed at the Pharmacy Laboratory of the Federal University of Paraíba (João Pessoa, Paraíba, Brazil). The samples without EDTA were centrifuged and sent to the Veterinary Clinical Laboratory of the Veterinary Hospital of the Federal University of Viçosa, for analysis of the biochemical parameters of the serum, using an automated clinical chemistry analyzer (HumaStar 300; Gesellschaft für Human Biochemica e Diagnostica MBH, Wiesbaden, Germany). The analysis was performed to detect the concentrations of urea, creatinine, alkaline phosphatase (ALP), alanine transaminase (ALT), and aspartate transaminase (AST).

### Statistical analysis

For data analysis, all animals were considered to be adults and we did not differentiate between male and female animals. The data were analyzed using SPSS 20.0 for Windows (IBM Corp., NY, USA). The hematology and blood biochemistry data did not present a normal distribution and were, therefore, analyzed using non-parametric statistics (Mann–Whitney). The level of significance was set at <5%.

## Results

Tables-1 and 2 present the mean and 95% confidence intervals for hematology and serum chemistry values in the TO and RT sample populations. Due to failures in logistics, four of the plasma samples from TO sloths were lost, and three of the samples from RT sloths were lost. Among all the animals, there was only one female in the TO group and two females in the RT group. Due to the small proportion of females, the data were not analyzed by sex.

**Table-1:** Mean, SD, minimum, maximum and p-value for the hematological variables found for brown-throated sloths in the urban populations of TO (n=8) and RT (n=8).

Parameters	Unit	Mean±SD TO	Minimum TO	Maximum TO	Mean±SD RT	Minimum RT	Maximum RT	p-value
Erythrocytes	million/mm^3^	3.22±0.14	3.02	3.42	3.05±0.18	2.79	3.30	0.17
Hemoglobin	g/dL	11.86±0.62	10.8	12.5	13.03±2.89	11.2	20.0	0.35
Hematocrit	%	34.8±1.82	32.1	37.0	38.57±7.48	33.2	55.7	0.25
Leukocytes	/mm^3^	8616.66±1235.71	6700	9700	13062.5±3684.30	7800	17700	0.01*
MCH	pg	36.78±1.38	35.0	38.8	37.26±2.11	33.8	40.2	0.64
MCV	µ^3^	107.88±4.26	101.8	113.7	110.12±3.90	103.8	116.1	0.32
MCHC	%	34.1±0.55	33.5	35.0	33.78±1.01	32.5	35.9	0.51
Platelets	mm^3^	60833.33±28017.25	26000.0	95000	38428.57± 19990.47	16000	73000	0.06
Neutrophils	%	22.37±12.43	12.0	23.0	21.57±4.50	15.0	27.0	0.23
Eosinophils	%	2±1.77	0.0	4.0	2±2.30	0.0	7.0	0.89
Lymphocytes	%	70.5±12.97	64.0	83.0	72±7.48	63.0	84.0	0.70
Monocytes	%	4.12±3.56	1.0	12.0	3.71±3.54	0.0	10.0	0.65

* Significantly different. SD=Standard deviation, TO=Teófilo Otoni, RT=Rio Tinto, MCH=Mean corpuscular hemoglobin, MCV=Mean corpuscular volume, MCHC=Mean corpuscular hemoglobin concentration

**Table-2:** Mean, SD, minimum, maximum, and p-value for the biochemical variables found for brown-throated sloths in the urban populations of TO (n=4) and RT (n=8).

Parameters	Unit	Mean±SD TO	Minimum TO	Maximum TO	Mean±SD RT	Minimum RT	Maximum RT	p-value
Urea	mg/dL	32±8.52	22.0	42.0	24.25±8.37	10.0	36.0	0.16
Creatinine	mg/dL	0.52±0.15	0,4	0,7	0.65±0.13	0.5	0.8	0.16
ALT	Ul/L	†	†	†	19.85±10.85	8.0	40.0	-
AST	Ul/L	205.75±25.15	181.0	239.0	209.75±68.68	131.0	380.0	0.91
ALP	Ul/L	280.50±48.48	233.0	343.0	443.33±217.04	239.0	810.0	0.22

† The analysis was not performed. SD=Standard deviation, TO=Teófilo Otoni, RT=Rio Tinto, ALT=Alanine transaminase, AST=Aspartate transaminase, ALP=Alkaline phosphatase

Brown-throated sloths in both municipalities had similar hematological levels, except for the total number of leukocytes, which were higher in brown-throated sloths from the RT group. Regarding the biochemical values of urea, creatinine, AST, and ALP, there were no significant differences between the brown-throated sloths from RT and TO. Biochemical comparisons of ALT were not performed due to a failure in processing the plasma aliquots of the TO group.

## Discussion

The main objective of this work was to establish standards of hematological and biochemical values for free-range brown-throated sloths in urban areas. Apart from total leukocytes, which were significantly higher in brown-throated sloths in the RT than in the TO group, the hematological and biochemical values were similar between the two subpopulations studied.

Elevations in the leukocyte count may generally occur because of chronic stress or disease [16]. The brown-throated sloths, in our study, were apparently healthy, with no clinical signs of disease such as changes in skin appearance and color, bedsores, pustules, abnormal lumps, or the presence of ectoparasites. All animals showed resistance to containment, suggesting normal reactivity, and the capture was an acute stress; however, both factors could not explain the change in leukocyte concentration. However, considering the data presented by other authors [17,18], there is a great variation in the reported leukocyte values in the studies (Table-3). The wide range of mean leukocyte concentration in brown-throated sloths in the RT group resulted in a high standard error, suggestive of some outliers in the samples. Indeed, some animals showed peak levels of 17,700 cells/mm^3^ (third quartile), while the maximum concentration in the TO group was 9700 cells/mm^3^ (second quartile). Therefore, when in the absence of disease or chronic stress, an idiosyncratic effect is expected on the leukocyte concentration, such as is seen in the sloth subpopulations from RT and TO.

**Table-3:** Comparison of the hematological variables found for the two populations of urban areas of brown-throated sloths with the literature [17], n=13 individuals; [23], n=25 individuals.

Parameters	Unit	Mean±SD TO	Mean±SD RT	Mean±SD [17]	Mean±SD [23]
Erythrocytes	million/mm^3^	3.22±0.14	3.05±0.18	4.20±0.72	3.20±0.53
Hemoglobin	g/dL	11.86±0.62	13.03±2.89	12.1±1.83	11.31±1.48
Hematocrit	%	34.8±1.82	38.57±7.48	35±3.99	35.60±3.24
Leukocytes	/mm^3^	8616.66±1235.71	13062.5±3684.30	6800.0±664	12960±612
Basophils	%	0.0	0.0	1.0±0.91	§
MCH	pg	36.78±1.38	37.26±2.11	§	35.50±7.82
MCV	µ^3^	107.88±4.26	110.12±3.90	§	113.90±21.28
MCHC	%	34.1±0.55	33.78±1.01	§	32.03±3.60
Platelets	mm^3^	60833.33±28017.25	38428.57±19990.47	§	23582±12755
Neutrophils	%	22.37±12.43	21.57±4.50	41.0±6.34	9.17±6.88
Eosinophils	%	2±1.77	2±2.30	6.0±2.34	3.22±2.24
Lymphocytes	%	70.5±12.97	72±7.48	49.0±7.35	55.88±25.09
Monocytes	%	4.12±3.56	3.71±3.54	3.0±1.29	2.11±1.94

§ Values did not present by the authors. SD=Standard deviation, TO=Teófilo Otoni, RT=Rio Tinto, MCH=Mean corpuscular hemoglobin, MCV=Mean corpuscular volume, MCHC=Mean corpuscular hemoglobin concentration

The observed values were similar between the two subpopulations of brown-throated sloths for concentrations of urea, creatinine, AST, and ALP (Table-2). It was not possible to analyze ALT in the TO group, and thus, the values for this enzyme, in this study, are only from the RT group. Similarities in other enzyme concentrations suggest a physiological response in brown-throated sloths specific to the species. Alternatively, liver overload as such as toxic dietary compounds (e.g., highest ingesting of mature *Ficus* leaves) could elicit the same metabolic responses in both TO and RT brown-throated sloths. Indeed, some of us have observed a high intake of mature leaves by TO and RT sloths [6,19].

Divergence was verified in relation to the enzyme values (Tables-3 and 4) described by other authors [17]. While the values of urea and creatinine were similar, the values of AST and ASP in the brown-throated sloths in our study were higher compared to the sloths in the other study [17].

**Table-4:** Comparison of the biochemical variables found for the two populations of urban areas of brown-throated sloths with the literature [17] (n=13 individuals).

Parameters	Unit	Mean±SD TO	Mean±SD RT	Mean±SD [17]
Urea	mg/dL	32±8.52	24.25±8.37	17.0±6.2
Creatinine	mg/dL	0.52±0.15	0.65±0.13	0.8±0.3
ALT	Ul/L	†	19.85±10.85	76.0±20.1
AST	Ul/L	205.75±25.15	209.75±68.68	85.0±27.4
ALP	Ul/L	280.5±48.84	443.33±217.04	31.0±21.3

† The analysis was not performed. SD=Standard deviation, TO=Teofilo Otoni, RT=Rio Tinto, ALT=Alanine transaminase, AST=Aspartate transaminase, ALP=Alkaline phosphatase

The ALT of RT brown-throated sloths was 3.8 times higher than that of urban sloths from a place in the state of Rio de Janeiro [17]. Compared to the same study [17], the ASP was 2.4 times higher and ALP was 9.0. times higher than that in Rio de Janeiro urban brown-throated sloths. High levels of ALT and AST can indicate liver disorders or inflammation [20]. ALT and AST occur in the cytoplasm of hepatocytes and are the best markers of hepatic infections. Increased ALP without other hepatic markers in young animals is suggestive of metabolic alterations in the bone and muscle tissue [20]. However, if the increase in ALP is matched by a simultaneous increase in the ALT levels, liver damage is a stronger clinical suspicion [20].

Toxic compounds can cause liver changes in animals without necessarily causing jaundice or other disease symptoms [20]. Changes in AST, ALT, and ALP concentrations may occur because to chronic toxicity of secondary compounds in the animals’ diet. Brown-throated sloths from TO and RT eat leaves of *Ficus* [6,21], which is only one genus of tree available in the squares. The leaves of *Ficus* sp. were described to correspond only to 19.51% of the brown-throated sloth’s natural diet, which is supplemented with other trees [22]. The leaves eaten by sloths may contain secondary compounds, which slows the digestion in animals. It is believed that due to the lower levels of secondary compounds, the sloth feeds more on young leaves [1,22]. Diet restriction for only one tree species can increase the competition for food in urban and restricted areas, resulting in a shortage of young leaves. In this scenario, the brown-throated sloths overeat mature leaves, which have more toxic compounds, resulting in liver overload.

The values obtained in this study should be interpreted with caution, when compared to those of the previous studies [17,18]. Several methodologies have previously been used to determine the hematological and serum biochemical values in sloths of different species; this precluded direct comparisons between most studies. However, two articles, in particular, presented hematological and biochemistry values for the same species, *B. variegatus* [17,18]. The first study [17] was performed in an urban square in Valença, Rio de Janeiro, localized in the Atlantic Forest biome, but the sloths were anesthetized with ketamine before venipuncture, in contrast to the present study. Another study [18] was carried out in the urban woods in Belém, a city in the Amazonian biome, where the climate and vegetation are very different from the Atlantic Forest biome.

Although our study aimed to describe the hematological and biochemical parameters of urban free-range brown-throated sloths, the data refer mostly to males because few females were captured. Therefore, caution should be exercised if a physiological or clinical evaluation considering the sex of the sloths is needed. There are similar challenges to living in squares or small woods in urban areas such as pollution, human disturbance, and low vegetal diversity. Therefore, the hematology and blood biochemistry data presented in this study could be considered as clinical reference indices for adult brown-throated sloths living in urban spaces.

## Conclusion

In this study, the leukocyte concentration showed a wide physiological range, probably resulting from an idiosyncratic effect in brown-throated sloths. High hepatic enzyme levels led to the suspicion of liver disorders in sloths from both RT and TO. The descriptions of hematological and biochemical profiles for brown-throated sloths are scarce, making comparison difficult. The current study is a source of clinical reference data for urban sloths living in cities of the Atlantic Forest biome.

## Authors’ Contributions

KFP: Background theory, design of experiment, methodology, data collection, data analysis and interpretation, writing and reviewing the text. IOS and VB: Coordinator, background theory, design of experiment, methodology, data collection, data analysis and interpretation, writing and reviewing the text. FFRS and VHDS: Methodology, data collection, data analysis and interpretation. CSSC: Coordinator, methodology, data collection, data analysis and interpretation, writing and reviewing of the text. All authors have read and approved the final manuscript.
